# KCC3a, a Strong Candidate Pathway for K^+^ Loss in Alkalemia

**DOI:** 10.3389/fcell.2022.931326

**Published:** 2022-07-07

**Authors:** Mohammed Zubaerul Ferdaus, Andrew Scott Terker, Rainelli Koumangoye, Eric Delpire

**Affiliations:** ^1^ Department of Anesthesiology, Vanderbilt University School of Medicine, Nashville, TN, United States; ^2^ Division of Nephrology, Department of Medicine, Vanderbilt University School of Medicine, Nashville, TN, United States

**Keywords:** K–Cl cotransport, intercalated cells, bicarbonate, metabolic alkalosis, K^+^ loss

## Abstract

Loss-of-function mutations in the human potassium chloride cotransporter-3 (KCC3) cause a hereditary motor sensory neuropathy associated with agenesis of the corpus callosum. While recapitulating the neuropathy, KCC3-knockout mice also exhibit high blood pressure. This phenotype is believed to have neurogenic and/or vascular origins. The role of KCC3 in the kidney is poorly understood. KCC3 is encoded by two major isoforms originating from alternative promoters: KCC3a and KCC3b, with KCC3b being the predominant transcript in the kidney. Although the transporter has previously been localized to the proximal tubule, we show here the unique expression of the KCC3a isoform in the connecting tubule. Using a KCC3a-specific polyclonal antibody validated for both immunofluorescence and immunoblotting, we showed an intense KCC3a signal restricted to cortical intercalated cells. No overlap is detected between KCC3a and sodium chloride cotransporter (NCC), a distal convoluted tubule (DCT) marker; or between KCC3a and ENaC or calbindin, which are both principal cell markers. KCC3a signal was observed in cells expressing the apical V-ATPase and pendrin, establishing a unique expression pattern characteristic of intercalated cells of type-B or type-nonA/nonB. We further show that treatment of wild-type mice with hydrochlorothiazide, amiloride, or fed a K^+^-deficient diet up-regulates KCC3a level, suggesting that volume depletion increases KCC3a abundance. This hypothesis was confirmed by showing a higher abundance of KCC3a protein after 23-h water restriction or after placing the mice on a low-salt diet. More importantly, abundance of the Cl^−^/HCO_3_^−^ exchanger, pendrin, which is known to secrete bicarbonate in alkalotic conditions, was significantly diminished in KCC3-knockout mice. In addition, KCC3a abundance increased significantly alongside pendrin abundance in bicarbonate-treated alkalotic mice, providing a credible mechanism for K^+^ loss in metabolic alkalosis.

## Introduction

Alkalemia is defined as an arterial blood pH exceeding a value of 7.45. It can be caused by the accumulation of alkali (HCO_3_^−^) or the loss of acids (H^+^) (Foy and De Morais, 2017; Emmett, 2020; Brinkman and Sharma, 2022). Different physiological conditions resulting in an increased intracellular shift of H^+^, gastrointestinal loss, or renal H^+^ waste lead to metabolic alkalosis. The consumption of alkali, the uses of diuretics, repeated vomiting, severe dehydration, and certain endocrine disorders also cause metabolic alkalosis (Berend, 2018; Emmett, 2020). The kidney plays a key role to neutralize metabolic alkalosis by rapidly excreting excess HCO_3_^−^ in the urine. One key mechanism for abating alkalosis is the secretion of bicarbonate by type-B intercalated cells in the distal nephron (Wall et al., 2020). This is achieved by pendrin, an exchanger located at the apical membrane of intercalated cells which secrete HCO_3_^−^ in exchange for Cl^−^ (Royaux et al., 2001). Loss of blood K^+^ is often associated with metabolic alkalosis. Hypokalemia and metabolic alkalosis are, for instance, observed in conditions like Bartter and Gitelman syndromes (Bhandari and Turney, 1998). In these cases, an increased Na^+^ delivery to the aldosterone-sensitive distal nephron results in the activation of the renal outer medullary potassium channel (ROMK), and subsequent loss of K^+^. Low blood K^+^ has also been linked to a reduction in pendrin level, which could contribute to the maintenance of alkalosis in hypokalemia (Xu et al., 2017). Pendrin’s ability to secrete HCO_3_^−^ in the urine depends on Cl^−^ recycling between the tubular lumen and the intracellular compartment. Different transport systems have been implicated in coupling Cl^−^ recycling to pendrin function along the distal nephron. The sodium-driven chloride/bicarbonate exchanger (NDCBE) and the cystic fibrosis transmembrane conductance regulator (CFTR) are two hypothesized examples of distal nephron transport systems that have been linked to pendrin activity (Wall, 2022).

KCC3, a K–Cl cotransporter mediating the tightly coupled efflux of K^+^ and Cl^−^, is expressed in the kidney (Pearson et al., 2001). KCC3 is involved in the pathogenesis of a human disease called HSMN/ACC (hereditary sensory-motor neuropathy associated with agenesis of corpus callosum) (Howard et al., 2002). The disorder was first reported in the medical literature by Dr. Frederick Andermann, a Canadian neurologist whose name is often associated with the syndrome (Andermann et al., 1972). A relatively high incidence of HSMN/ACC cases exists in parts of Quebec, Canada, due to a founder effect dating back to the 17th century (Andermann and Andermann, 1994). In addition, there are additional sporadic cases described in the literature (Boettger et al., 2003; Uyanik et al., 2006). While mouse models of inactive KCC3 recapitulate the neuropathy phenotype, the mice also display high blood pressure (Boettger et al., 2003; Adragna et al., 2004; Garneau et al., 2016). Studies have attributed this high blood pressure to neurogenic (Rust et al., 2006) and vascular effects (Adragna et al., 2004). The possibility that renal KCC3 might also be involved in the blood pressure phenotype has yet to be addressed.

The human protein atlas reveals low RNA tissue specificity for KCC3 with relatively high expression levels in the retina, bone marrow, testis, vagina, esophagus, kidney, and lower levels in all other tissues (Uhlén et al., 2015; Sjöstedt et al., 2020). In 2001, we reported two distinct transcripts of KCC3 starting with alternative first exons: KCC3a starting with exon 1a (encoding 90 amino acids) and KCC3b, initiated from exon 1b (encoding 39 distinct amino acids). The KCC3a transcript is more abundant in the brain, whereas KCC3b is more abundant in the kidney (Pearson et al., 2001). RNA-seq analyses of microdissected rat kidney tubule segments reveal a low expression level of KCC3 along the nephron (Lee et al., 2015). The analysis, however, does not distinguish between the two major isoforms. Information about KCC3 expression and function in the kidney is rather limited. Using an antibody made to an exon 3–specific peptide, we showed KCC3 at the basolateral membrane of the proximal tubule S1 segment (Mercado et al., 2005), a signal later shown to increase in hyperglycemia (Melo et al., 2013). Another study examining renal function in the KCC3-knockout mouse revealed increased diuresis (Garneau et al., 2016). Interestingly, aldosterone was unchanged despite a significant increase in mean arterial pressure and blood K^+^, indicating possible renal involvement in the observed phenotype.

Here, we show abundant expression of KCC3a in intercalated cells type-B or type-nonA/nonB. The signal was most abundant in the cortex, indicating expression in connecting tubule (CNT), the aldosterone-sensitive segment that links the distal convoluted tubule to the collecting duct. Furthermore, we discovered that KCC3a and pendrin are expressed in the same cells and are functionally connected in terms of HCO_3_^−^ secretion. We propose that coupling of pendrin function with KCC3a function results in KHCO_3_ secretion and that KCC3a might be a key pathway for K^+^ loss in metabolic alkalosis.

## Materials and Methods

### Animal Experiments

C57BL/6J male and female mice aged 2–3 months were used in this study. All animals had been maintained in a temperature-controlled and pathogen-free barrier facility in ventilated cages with a 12-h light/12-h dark cycle. KCC3-knockout (KCC3-KO) mice were generated by disrupting exon 3 using targeted homologous recombination in the embryonic stem cells as described before (Howard et al., 2002). Homozygous KCC3-KO (KCC3^−/−^) and controls (KCC3^+/+^) were generated by breeding heterozygous KCC3 (KCC3^+/−^) mice. For diet manipulation, mice were maintained on control diet (Envigo Teklad custom diet, TD.88238; 1.05% K^+^, 0.29% Na^+^, 0.9% Cl^−^), potassium-deficient diet (Envigo Teklad custom diet, TD.88239; 0% K^+^, 0.29% Na^+^, 0.45% Cl^−^), high-sodium diet (Envigo Teklad custom diet, TD.190009; 1.05% K^+^, 1.57% Na^+^, 3.38% Cl^−^), or sodium-deficient diet (Envigo Teklad custom diet, TD.190152; 1.05% K^+^, 0 Na^+^, 0.95% Cl^−^) for 4–5 days with free access to water. For the water deprivation study, C57BL/6J mice were randomly assigned to 23-h water deprivation with free access to food, while control mice had free access to both water and food. For tissue collection, mice were sacrificed by isoflurane administration (inhalation), followed by cervical dislocation. All studies using mice were approved by the Vanderbilt Animal Care and Use Committee.

### PCR Genotyping

Mice were genotyped at age ∼postnatal day 21 by collecting tail snips under isoflurane anesthesia. The 3–5 mm tail samples were digested in 200 μL lysis buffer (25 mM NaOH, 0.2 mM EDTA, pH ∼12.0) at 95°C for 1 h and then neutralized with 200 μL Tris buffer (40 mM, pH ∼5.0). PCR reactions were set up to identify the mutant gene (forward: 5′ GAA​CTT​TGT​GTT​GAT​TCC​TTT​GG 3′; reverse: 5′ TAC​AAC​ACA​CAC​TCC​AAC​CTC​CG 3′) and a control gene (forward: 5′ GAA​CTT​TGT​GTT​GAT​TCC​TTT​GG 3′; reverse: 5′ TCTCCTAACTCCA TCTCCAGGG 3’). PCR reactions were run on 1.5% agarose gel.

### Kidney Western Blot

Kidneys were snap-frozen in liquid nitrogen immediately after harvesting and utilized directly or stored at −80°C. Single kidneys were homogenized using a Potter homogenizer in 1 mL ice-cold homogenization buffer containing 300 mM sucrose, 50 mM Tris-HCl (pH 7.4), 1 mM EDTA, 1 mM EGTA, 1 mM dithiothreitol, 1 mM phenylmethylsulphonyl fluoride, Halt protease and phosphatase cocktail (Thermo Fisher Scientific, catalog#: 78442), and PhosSTOP phosphatase inhibitor cocktail tablet (Roche, catalog#: 04906837001). Homogenate was centrifuged at 6,000 rpm for 15 min at 4°C, and the supernatant was transferred to a new tube and stored at −80°C. Protein assay was done using Bio-Rad protein assay dye reagent (Bio-Rad, catalog#: 5000006). Then, 40 μg protein was separated on a 4–20% Mini-PROTEAN TGX Precast protein gel (Bio-Rad, catalog#: 4561095). Proteins were transferred to a polyvinylidene fluoride membrane using the Trans-Blot Turbo Transfer System (Bio-Rad, catalog#: 1704150). The membrane was blocked with 5% nonfat milk in TBS-Tween, followed by incubation with primary antibody for either 1 h at room temperature or overnight at 4°C. The membrane was then washed in TBS-Tween, incubated with horseradish peroxidase-conjugated anti-rabbit IgG (Promega, catalog#: W401B) at room temperature, and washed again in TBS-Tween. Proteins on the membrane were visualized by incubating them in luminol/enhancer solution (Bio-Rad Clarity Western ECL Substrate, catalog#: 1705061). The signal was detected with a ChemiDoc MP Imaging System (Bio-Rad, catalog#: 12003154). Protein signal quantification was performed with ImageJ (http://rsbweb.nih.gov/ij/) with all data normalized to actin.

### Immunofluorescence

Kidneys were fixed in 10% neutral buffered formalin and slides with paraffin-embedded sections were prepared. The tissues on slides were de-paraffinized and rehydrated in decreasing strengths of ethanol and then washed with PBS at room temperature. After antigen retrieval in citra plus solution (BioGenex, Catalog#: HK080-9K), tissue sections were incubated overnight at 4°C with primary antibodies (Table 1), followed by poly-HRP–conjugated secondary antibody (Table 1) for 30 min, washed in PBS and incubated with tyramide solution for 2 min at room temperature. After washing in PBS, tissue sections were mounted on microscope slides with ProLong Gold antifade reagent with DAPI (Invitrogen, catalog#: P36931). Images were captured with a Zeiss LSM 880 laser scanning confocal microscope.

**TABLE 1:** Antibodies.

Antibody	Host/Type	Dilution (use)	Source	References/Catalogue
KCC3a	Rabbit polyclonal	1:500 (WB), 1:100 (IF)	Eric Delpire	Ding and Delpire, (2014)
Pendrin	Rabbit	1:5000 (WB), 1:1000 (IF)	Susan Wall	(Knauf et al., 2001; Pham et al., 2020)
pNCC	Rabbit polyclonal	1:2000 (WB)	PhosphoSolutions	p1311-53
GFP	Alpaca	1:40 (IP)	ChromoTek	Gta-20
GFP	Mouse monoclonal	1:1000 (WB)	VAPR	Clone 1C9A5
pNCC-T46	Sheep polyclonal	1:50 (IF)	MRC Dundee	S241C
V-ATPase	Mouse monoclonal	1:100 (IF)	Santa Cruz	sc-55544
Calbindin-28	Mouse monoclonal	1:100 (IF)	Abcam	ab82812
ENaC (γ)	Rabbit polyclonal	1:50 (IF)	StressMarq	SPC-405D
β-actin	Rabbit monoclonal	1:3000 (WB)	Abcam	ab8227
anti-rabbit	HRP-conjugated	1:2000	Promega	W401B
anti-mouse	HRP-conjugated	1:2000	Promega	W402B

KCC3a, K^+^–Cl^-^ cotransporter isoform a; pNCC, phosphorylated NCC (Thr53); ENaC, epithelial sodium channel; V-ATPase, vacuolar-type ATPase; HRP-conjugated, horseradish peroxidase-conjugated; WB, Western blot; IF, immunofluorescence; VAPR, Vanderbilt Antibody and Protein Resource.

### KCC3a-Specific Antibody

The KCC3 antibody utilized in this study was raised in rabbits against the first 132 amino acids of mouse KCC3a. This fragment is encoded by exon 1a, exon 2, and the majority (84%) of exon 3. The KCC3a-specific antibody was then purified using a fusion protein that included only the first 44 residues of the transporter, encoded by part of exon 1a. To establish the specificity of the antibody, we created proteins with exon 1a and exons 2–3 fused to EGFP. Exons were PCR amplified from mouse cDNA and inserted into a pCDNA5 vector that contains the entire open reading frame of EGFP. After verification of proper sequence by sequencing, the DNA was purified with a Qiagen Medi prep kit and transfected into HEK293-T cells for expression.

### Cell Culture and Transfection

HEK293-T cells were maintained in the DMEM:F12 medium (Gibco, catalog#:11320-033), supplemented with 10% fetal bovine serum (R&D Systems, catalog#: S111110) and 2% penicillin/streptomycin (Gibco, catalog#: 15140-122). Cells in 10-cm dishes at 60–80% confluency were transfected with 15 μg DNA and 45 μL Fugene 6 transfection reagent (Promega, catalog#: 11 814 443 001). Briefly, 15 μg DNA was added to 400 μL Opti-MEM (Gibco, catalog#: 31985-070), followed by 45 μL Fugene 6. After vigorous mixing with a pipet, the mixture was incubated at room temperature for 20 min prior to dropwise addition to a 10-cm culture dish. Cells were then returned to the 37°C air with a 5% CO_2_ incubator for 36–48 h. Cells were then washed once with 10 mL Hank’s saline with calcium and magnesium (Gibco, catalog#: 14175-095), scraped with 700 μL RIPA buffer (Sigma, catalog#: R0278) containing protease inhibitors, incubated on ice for 20 min, briefly sonicated, incubated on ice for an additional 10 min, and centrifuged at 21.1 g for 20 min.

### Immunoprecipitation

Cell lysate (500 µg) was mixed with 25 μL GFP-Trap Agarose beads (ChromoTek, Planegg-Martinsried, Germany) in a 1.5-mL Eppendorf tube by rotating end-over-end for 2 h at 4°C. The bead–protein complex was washed three times in RIPA buffer and sedimented by centrifugation at 2,500 × g for 5 min at 4°C. Immunoprecipitates were resuspended in 80 μL of 4x Laemmli-sample buffer with dithiothreitol and incubated at 65°C for 15 min to dissociate immunocomplexes from beads. The beads were sedimented by centrifugation at 2,500 x g for 2 min at 4°C. Precipitated proteins in the supernatant were separated by SDS-PAGE and blotted on PVDF membranes. Membranes were probed with the primary antibody followed by HRP-conjugated secondary antibody. Proteins on the membrane were visualized by incubating them in the luminol/enhancer solution (Bio-Rad). The signal was detected with a ChemiDoc MP Imaging System.

### Diuretic Test

C57BL/6J mice were randomly assigned to a vehicle (glycol:water = 300:100), hydrochlorothiazide (37.5 mg/kg body weight) or amiloride (9.375 mg/kg body weight) treatment, administered once a day for 7 days by oral gavage. Blood and kidneys were collected after 7 days of treatment.

### Urine Analysis

Urine K^+^ was analyzed using the Diamond Diagnostics CareLyte Plus electrolyte analyzer. For urinary K^+^ measurement, a 100 μL urine sample was employed.

### Blood Analysis

Blood was collected *via* cardiac puncture and transferred into heparinized tubes. Immediately after collection, 80 μL of blood was loaded into a Chem8+ cartridge for electrolyte measurement using an i-STAT analyzer (Abbot Point of Care).

### Statistics

The null hypothesis was tested using 2-tailed unpaired t-tests or paired *t*-test by using GraphPad Prism 9 as indicated in the figure legends. All data are plotted as means ± SEM. *p* < 0.05 was considered significant.

## Results

### KCC3a Antibody Validation

**FIGURE 1:**
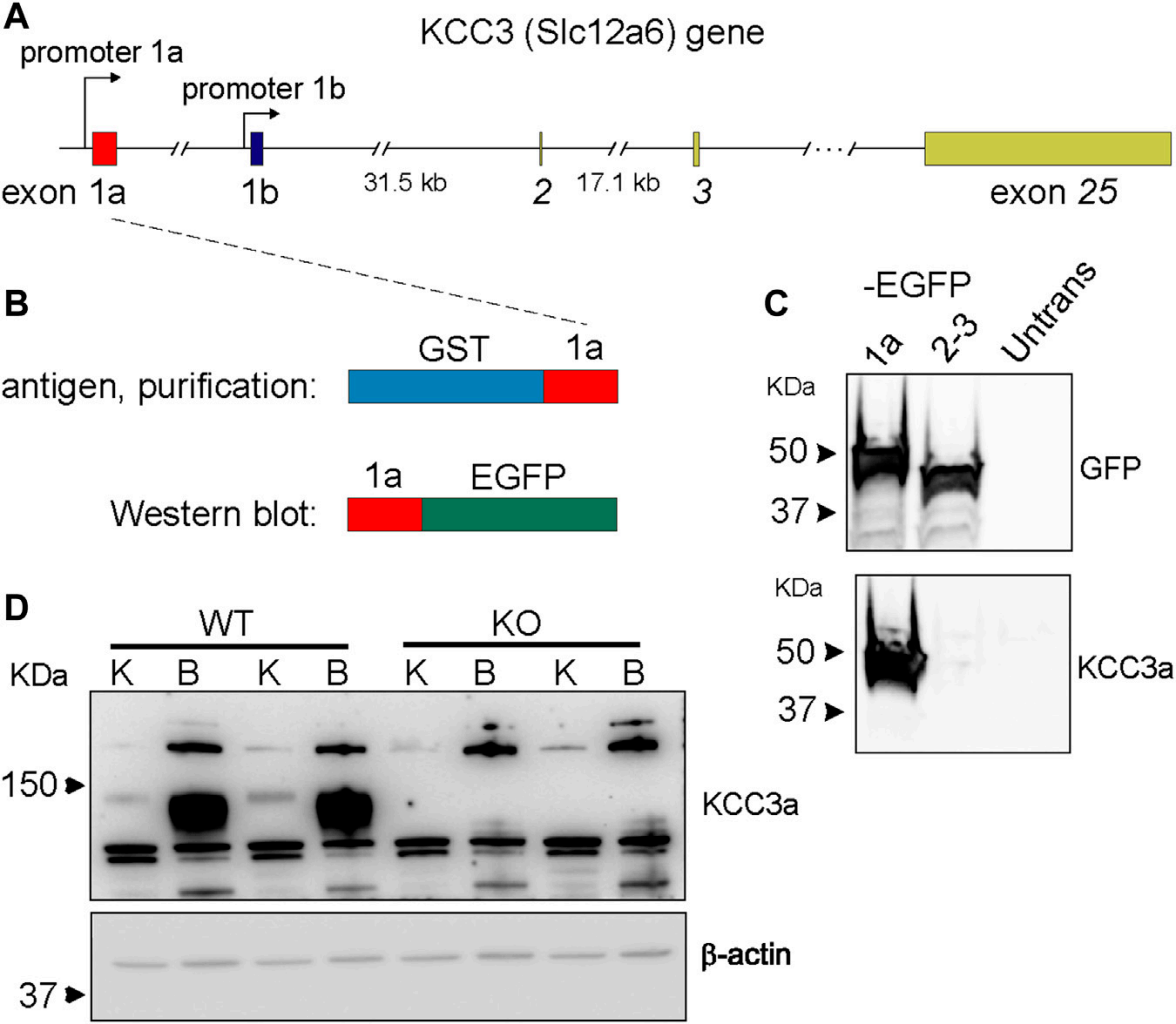
Generation of a potassium chloride cotransporter 3a (KCC3a)–specific antibody. **(A)** Structure of the mouse *Slc12a6* (KCC3) gene showing alternative promoters and exons 1a and 1b, as well as exons 2, 3, and 25. **(B)** Glutathione-S-transferases (GST)–exon 1a fusion protein used to purify the rabbit immune serum and fusion protein exon 1a–enhanced green fluorescent protein (EGFP) purified from transfected HEK293 cells used to assess antibody specificity. **(C)** Immunoblot showing EGFP expression in HEK293 cells transfected with 1a-EGFP or 2-3-EGFP but not untransfected cells. KCC3 signal in cells transfected with 1a-EGFP, but not 2-3-EGFP or untransfected cells. **(D)** Immunoblot shows the band at ∼130 kDa with kidney (K) and brain (B) samples from wild-type mice, but not from KCC3-knockout (KO) mice. Notably, there is an abundance of KCC3a in brain tissue, compared to the kidney.

The KCC3a antibody was generated by immunizing rabbits with a peptide fragment encoded by exon 1a–3 (Figure 1A) fused to glutathione-S-transferase (Ding and Delpire, 2014). The antibody was then purified from serum using a shorter GST fusion protein, *specific to exon 1a*, and validated by Western blot analysis using exon 1a-EGFP, a fusion protein expressed in HEK293-T cells (Figures 1B,C), as well as brain and kidney lysates isolated from wild-type mice and KCC3-knockout littermates (Figure 1D). In mouse tissues, the specific signal for KCC3a was observed at ∼130 kDa and absent from the KCC3-knockout mice. Notably, there was a relatively low abundance of the protein in kidney samples compared to brain samples.

### Localization of KCC3a Along the Nephron

**FIGURE 2:**
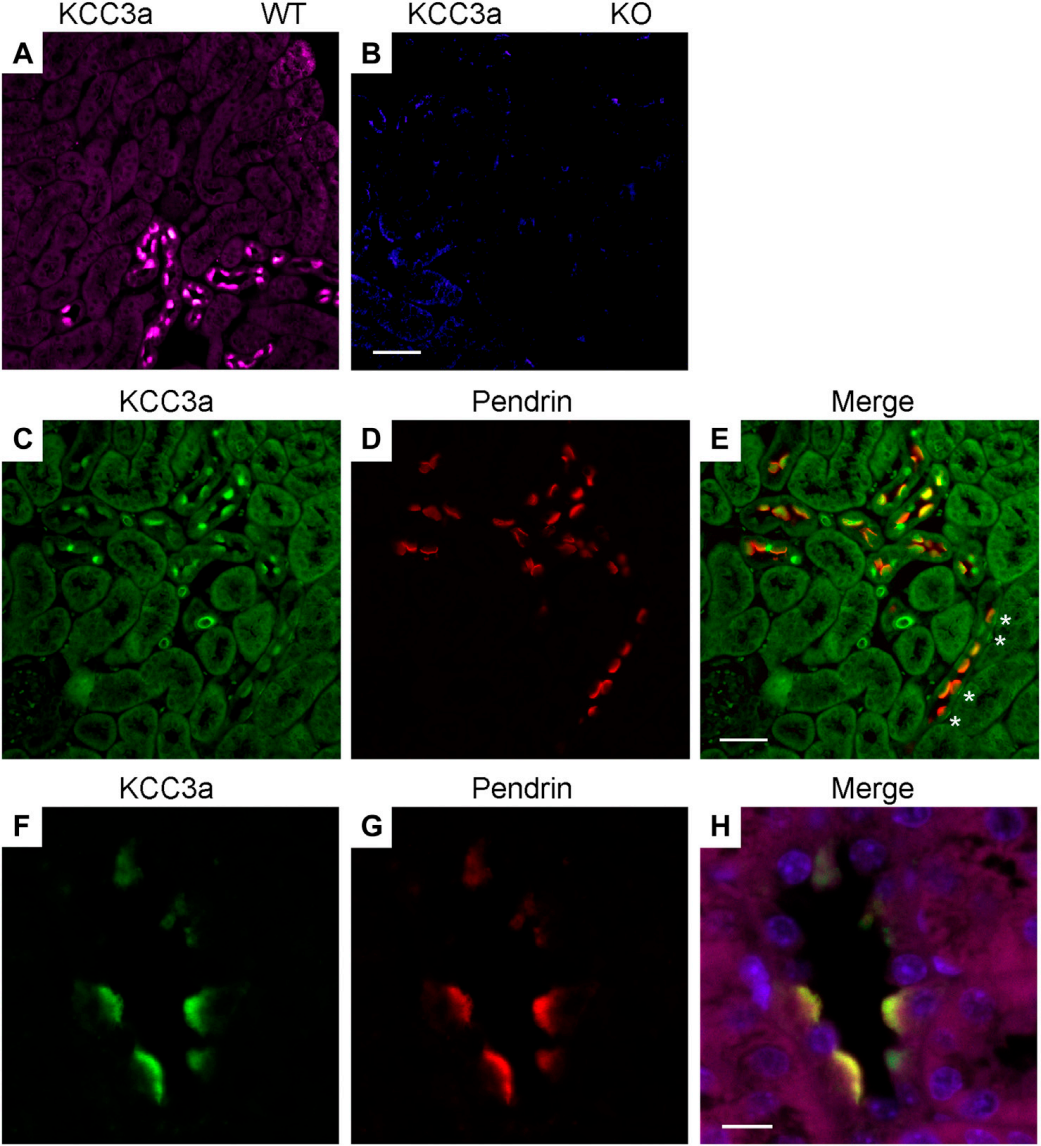
Potassium chloride cotransporter 3a (KCC3a) expression in pendrin-expressing cells. KCC3a signal (purple) in the kidney cortex of wild-type mice **(A)**, but not KCC3-knockout mice **(B)**. KCC3a expression [green, **(C)**] and pendrin expression [red, **(D)**] with an overlap of signal [yellow, **(E)**] in the kidney cortex at 40X. Notably, there is a presence of cells with faint basolateral KCC3a staining (white stars). Similarly, KCC3a expression [green, **(F)**] and pendrin expression [red, **(G)**] with overlap [yellow, **(H)**] at 63X. The absence of KCC3 expression in most tubule segments surrounding the positive cells is also noted. Scale bars = 50 µm for panels **(A–E)** and 10 µm for panels **(F–H)**.

**FIGURE 3:**
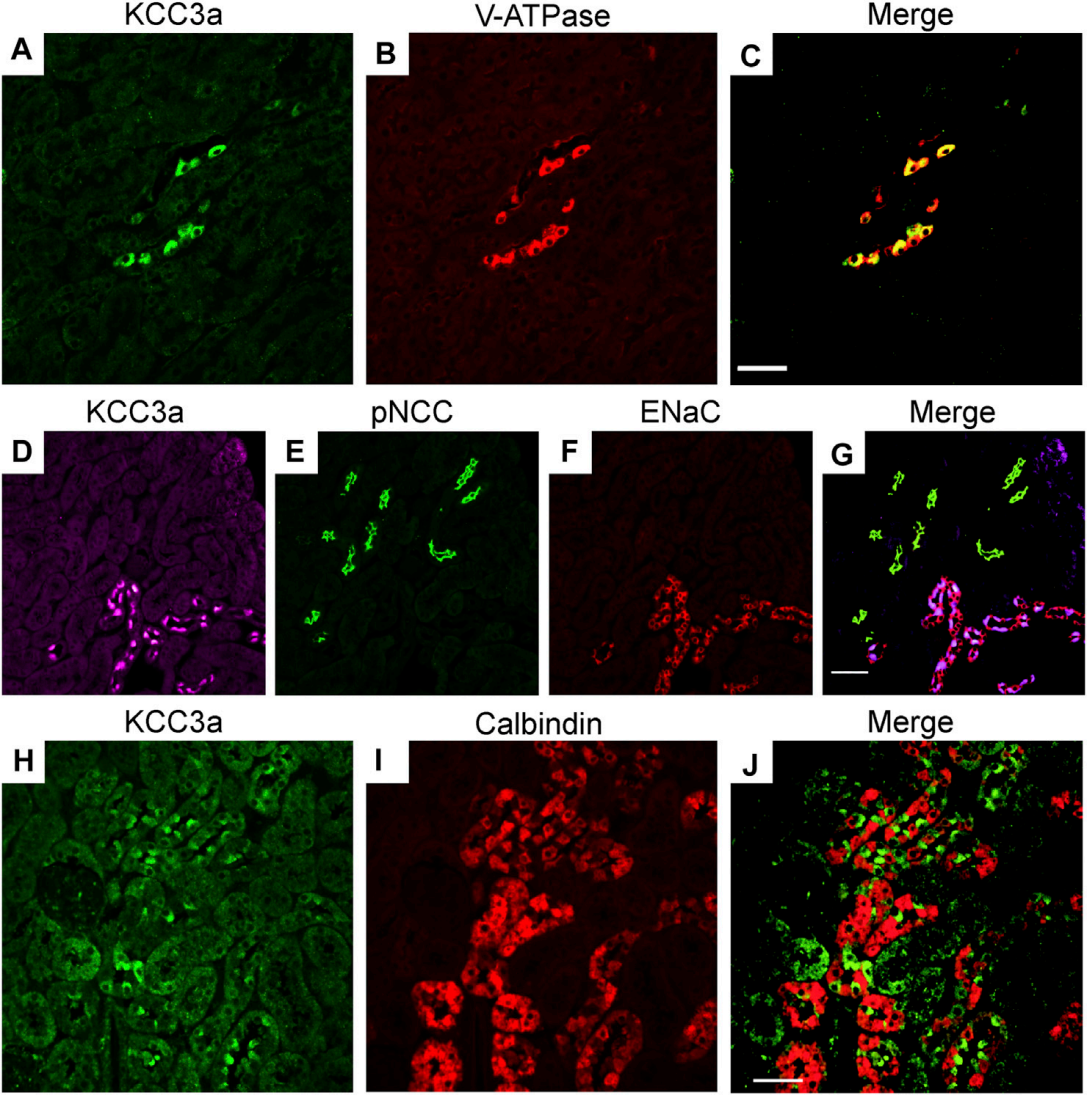
Potassium chloride cotransporter 3a (KCC3a) expression along the connecting tubule. KCC3a expression [green, **(A)**] and vacuolar-type ATPase (V-ATPase) expression [red, **(B)**] with overlap of signal [yellow, **(C)**] in kidney cortex. KCC3a expression [purple, **(D)**], phosphorylated sodium chloride cotransporter (pNCC) [green, **(E)**], epithelial sodium channels (ENaC) [red, **(F)**], and merged signal **(G)** in the kidney cortex. Expression of KCC3a [green, **(H)**], connecting tubule-specific principal cell marker—Calbindin-28 [red, **(I)**] and merged image **(J)** in the renal cortex. The signal for KCC3a is interspersed among the calbindin-positive cells. Scale bar = 50 µm.

Immunofluorescence (IF) was performed to determine the localization of KCC3a along the nephron and the antibody was validated for IF using KCC3-knockout mice. In kidney sections from wild-type mice, the KCC3a signal was generally low, except in distinct cells in the cortex. This signal was absent in KCC3-knockout mice (Figures 2A,B). The discontinuous pattern of expression indicated the presence of KCC3a in the CNT. Using co-staining with pendrin antibody (Figures 2C–E), an intercalated cell marker, we localized KCC3a in intercalated cells of type-B or type-nonA/nonB. While a faint signal could be seen in a few cells on the basolateral membrane (white stars, Figure 2E), most of the signal was apical. Figures 2F–H show higher magnification images with KCC3a and pendrin clearly overlapping at the apical pole. Further co-labeling experiments showed that the KCC3a signal was present in V-ATPase–positive cells (Figures 3A–C) and distinct from phosphorylated sodium chloride cotransporter (pNCC) (Figures 3D,E,G), ENaC (Figures 3D,F,G), and calbindin-28 signals (Figures 3H–J), indicating the absence of KCC3a expression in distal convoluted tubule (DCT, identified with pNCC) and CNT principal cells (identified with ENaC and calbindin-28). Notably, we did not confirm the absence of KCC3a expression in type-A intercalated cells.

### Changes in KCC3a Abundance in Response to Blood K^+^

**FIGURE 4:**
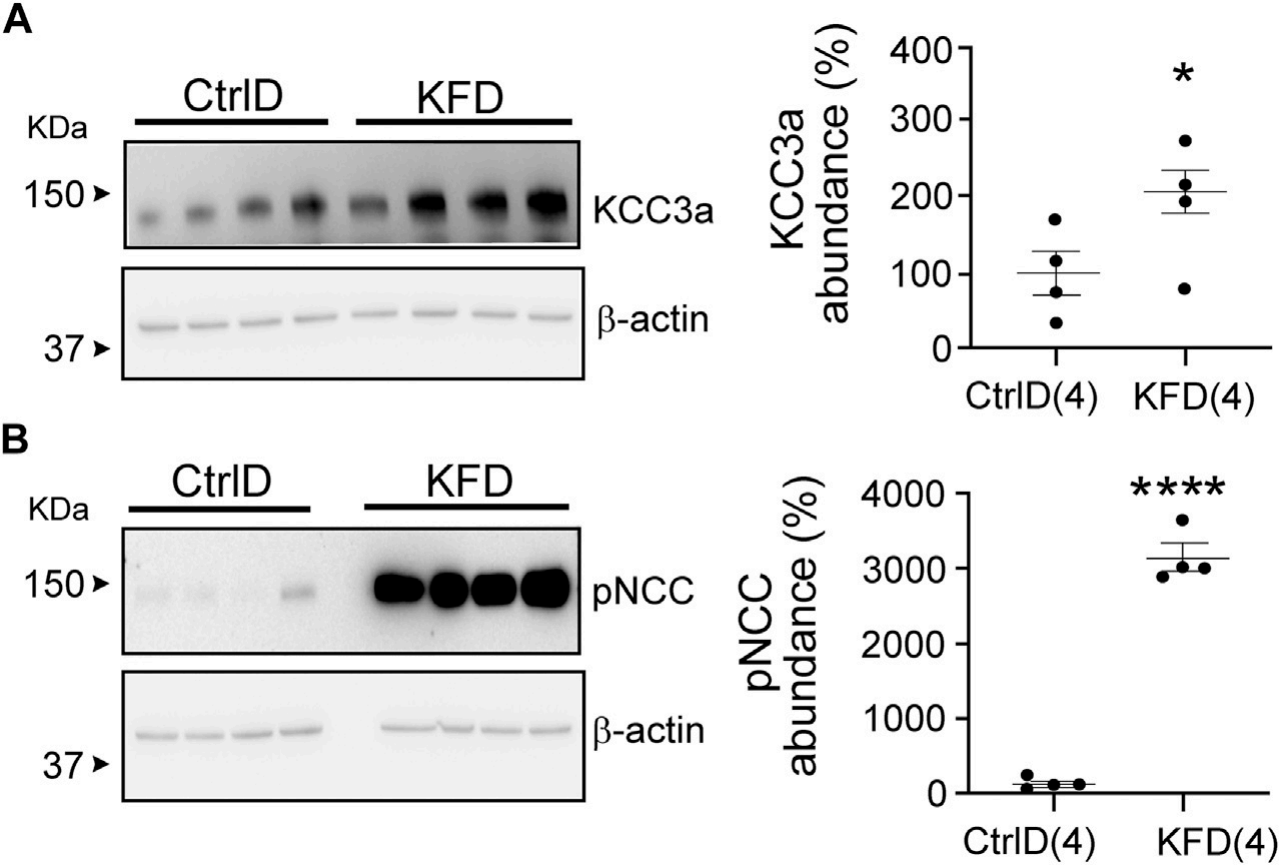
Change in potassium chloride cotransporter 3a (KCC3a) abundance in the kidney in mice on K^+^-deficient diet. **(A)** KCC3a abundance increased on K^+^-free diet (KFD) than in the control (Ctrl) diet (two-tailed unpaired *t*-test). **(B)** The phosphorylated sodium chloride cotransporter (pNCC) abundance was higher on the KFD than on the control diet (two-tailed unpaired *t*-test), indicating the diet’s efficiency. For blot quantification, densitometric values were normalized to β-actin. Values are means ± SEM; values in parentheses indicate n values. *p < 0.05; ****p < 0.0001.

Blood K^+^ is known to exert a strong effect on the distal nephron (Terker et al., 2015; Ferdaus et al., 2016; Terker et al., 2016). As KCC3a transports both K^+^ and Cl^−^, we investigated whether a change in blood K^+^ affects KCC3a expression. Mice were fed with a K^+^-deficient diet for 4–5 days to decrease their blood K^+^ concentration (López-Cayuqueo et al., 2018). KCC3a abundance was significantly increased after 4–5 days of dietary K^+^ deficiency, compared with mice maintained on a matched control diet (Figure 4A). Signal quantitation showed 100% ± 29.10 for control K^+^ diet *vs* 207.3% ± 28.01 for K^+^ free diet (values are mean ± SEM, *p* = 0.03). To confirm the effectiveness of the K^+^-deficient diet, we analyzed pNCC abundance (Figure 4B), which showed a significant increase in pNCC signal in the K^+^-free diet group (100% ± 37.98 for control K^+^ diet *vs.* 3035% ± 169.3 for K^+^-free diet, *p* < 0.0001).

**FIGURE 5:**
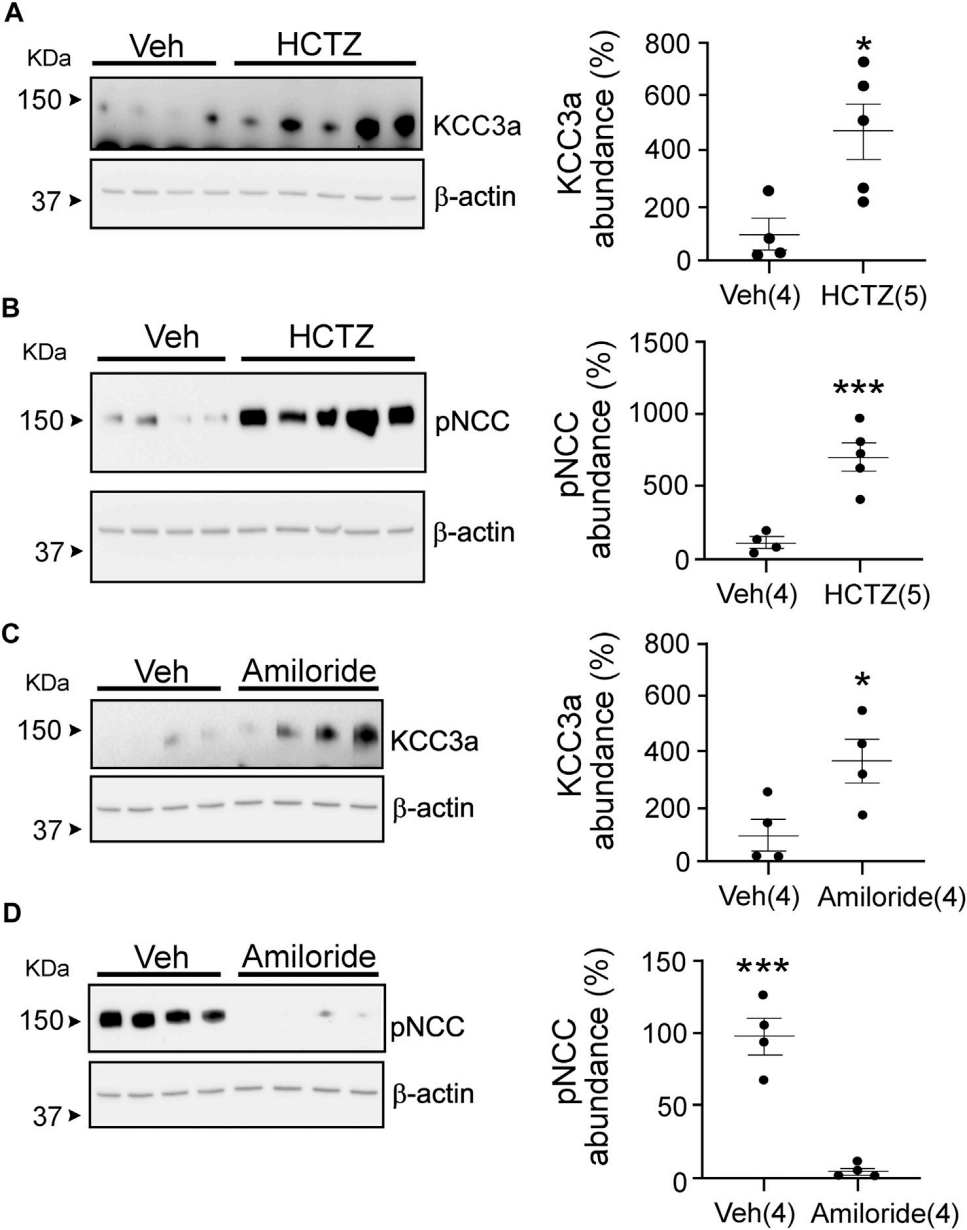
Potassium chloride cotransporter 3a (KCC3a) abundance changes in response to hydrochlorothiazide (HCTZ) treatment. **(A)** KCC3a abundance increases after HCTZ (37.5 mg/kg body weight) treatment compared with the vehicle (Veh, glycol:water = 300:100)-treated group (two-tailed unpaired *t*-test). **(B)** After HCTZ treatment, compared with the Veh-treated mice, the abundance of phosphorylated sodium chloride cotransporter (pNCC) increases (two-tailed unpaired *t*-test), demonstrating the drug’s effectiveness. **(C)** The abundance of KCC3a in amiloride-treated mice was higher than in vehicle-treated mice (two-tailed unpaired *t*-test). **(D)** In mice treated with vehicle, the abundance of pNCC was lower than in mice treated with amiloride (two-tailed unpaired *t*-test). For blot quantification, densitometric values were normalized to β-actin. Values are means ± SEM; values in parentheses indicate n values. *p < 0.05; ***p < 0.001.

Hydrochlorothiazide and amiloride treatments are known to cause opposite effects on blood K^+^ (Table 2), with a decrease in K^+^ associated with NCC inhibition (Gamba et al., 1993; Monroy et al., 2000; Terker et al., 2015). Interestingly, KCC3a abundance increased in mice treated for seven days with either hydrochlorothiazide or amiloride (Figures 5A,C). Indeed, KCC3a signal was 100% ± 54.73 in mice treated with vehicle *vs.* 467.6% ± 100.5 for mice treated with hydrochlorothiazide (*p* = 0.02) and 100% ± 60.38 for vehicle *vs.* 367.4% ± 85.97 for mice treated with amiloride (*p* = 0.04). Again, the effect of the diuretic treatments was assessed using pNCC abundance. Increased pNCC was observed in hydrochlorothiazide-treated mice (Figure 5B, 100% ± 34.0 for vehicle *vs.* 710.7% ± 96.65 for hydrochlorothiazide, *p* = 0.001). Conversely, decreased pNCC abundance was observed in mice treated with amiloride (Figure 5D, 100% ± 12.91 for vehicle *vs.* 2.87% ± 2.05 for amiloride, *p* = 0.0003).

**TABLE 2:** Blood [K^+^].

Vehicle mean ± SEM, mmol/L	HCTZ mean ± SEM, mmol/L	Amiloride mean ± SEM, mmol/L
3.4 ± 0.03	3.4 ± 0.16	6.4 ± 0.42**

HCTZ, hydrochlorothiazide; **p = 0.001 vehicle *versus* amiloride.

### Effect of Volume Depletion on KCC3a

**FIGURE 6:**
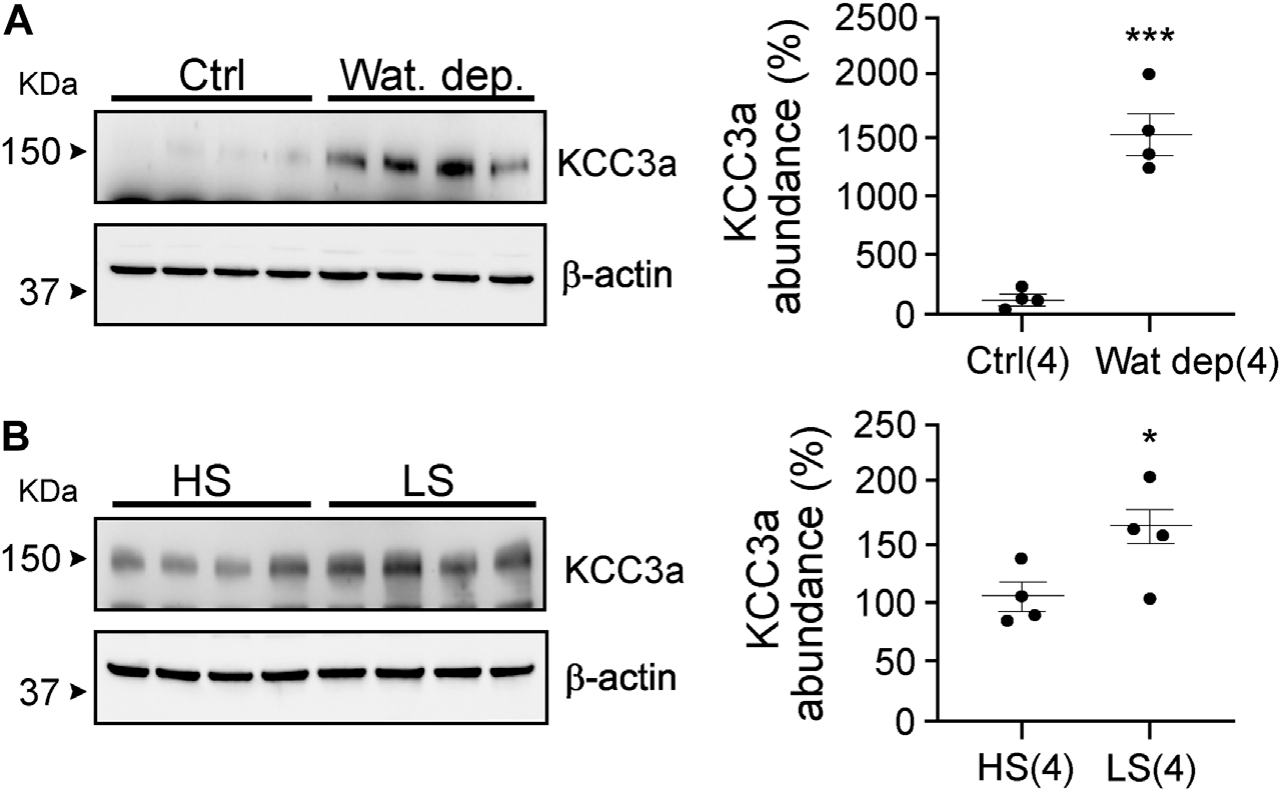
Potassium chloride cotransporter 3a (KCC3a) abundance changes in response to water deprivation and high-salt versus low-salt diets. **(A)** Mice were water restricted for 23 h, (Wat. Dep.) and KCC3a abundance was compared to mice maintained on water during the same period Ctrl, (two-tailed unpaired *t*-test). **(B)** Mice were fed for 4 days on a high-salt or low-salt diet and analyzed for KCC3a expression (two-tailed unpaired *t*-test). For blot quantification, densitometric values were normalized to β-actin. Values are means ± SEM; values in parentheses indicate n values. *p < 0.05; ***p < 0.001.

Our data with hydrochlorothiazide and amiloride treatments indicate that KCC3a abundance might not be following blood K^+^, but rather water loss and volume contraction. Indeed, both diuretics reduce the reabsorption of Na^+^ and water, leading to volume contraction. To address this possibility, we subjected mice to a 23-h water deprivation protocol and to salt restriction. As seen in Figure 6A, KCC3a abundance increased significantly in mice after 23 h of water deprivation compared to mice with free access to drinking water [100% ± 42.48 (control) vs. 1466% ± 171.1 (water deprivation), mean ± SEM, *p* = 0.0002]. Na^+^-deficient diet is known to cause volume depletion (Bagshaw et al., 2009; Bie, 2009). Again, as observed in Figure 6B, KCC3a abundance was significantly higher after four days on a low-Na^+^ diet compared to mice fed for four days on a high-Na^+^ diet [100% ± 12.25 (high-salt diet) *vs* 156.9% ± 13.57 (low-salt diet), *p* = 0.02].

### KCC3a Responds to Alkalosis

**FIGURE 7:**
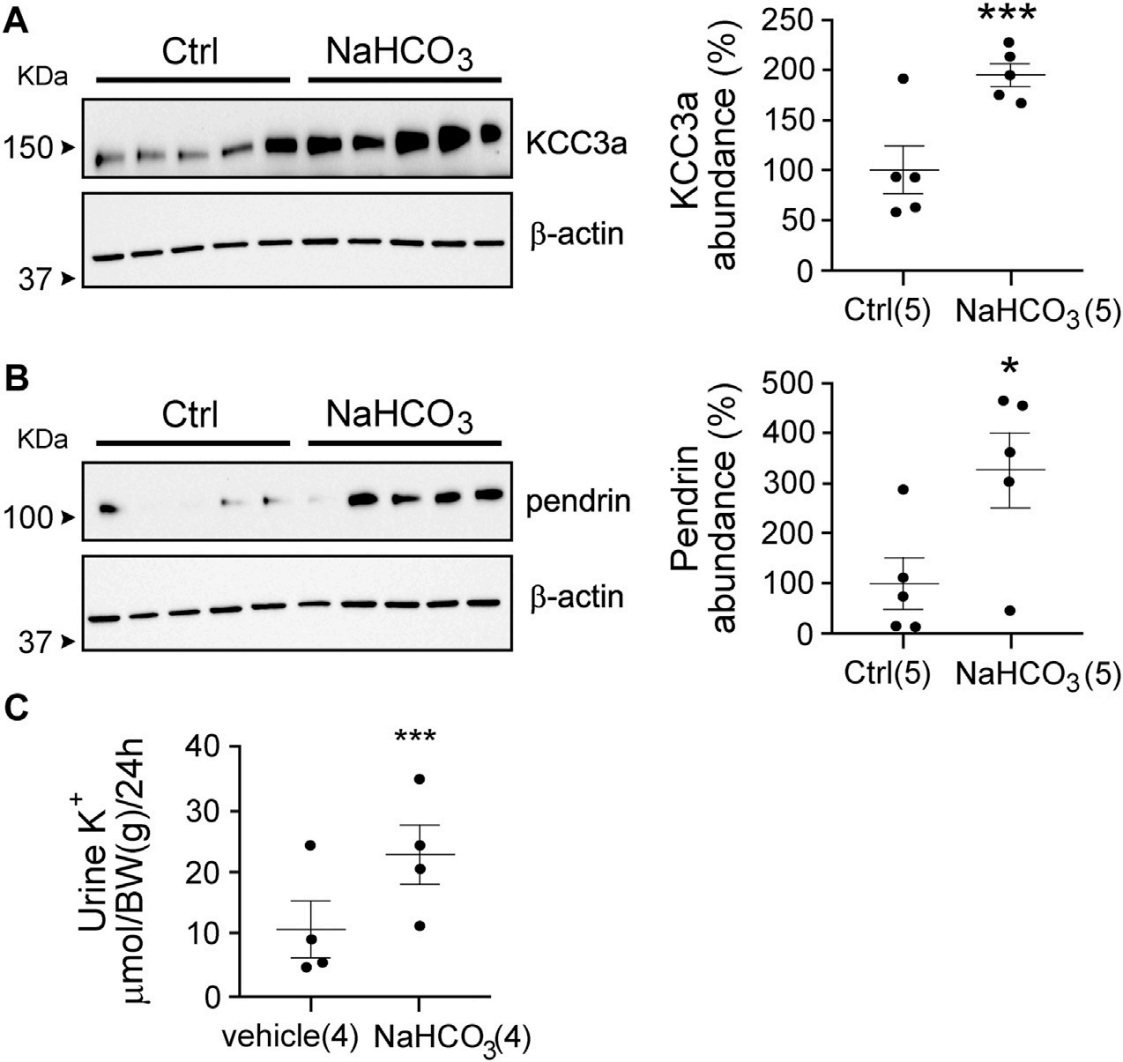
Link between pendrin and KCC3a. **(A,B)** NaHCO_3_ treatment for 24 h increased KCC3a and pendrin abundances compared to control (Ctrl), (two-tailed unpaired *t*-test). **(C)** Urinary K^+^ excretion was higher in NaHCO_3_-induced alkalotic condition (two-tailed paired *t*-test). For blot quantification, densitometric values were normalized to β-actin. Values are means ± SEM; values in parentheses indicate n values. *p < 0.05; ***p < 0.001.

Our observations suggest that maneuvers causing loss of water or volume depletion increased the abundance of the KCC3a protein. KCC3a localizes on the apical membrane of pendrin-expressing intercalated cells in the kidney cortex. To determine if KCC3a tracks with pendrin, we fed mice for 24 h with 280 mM bicarbonate in drinking water and observed a marked increase in both KCC3a and pendrin abundance (Figures 7A,B). For KCC3a, the increase was 2-fold [100% ± 24.03 (sucrose) *vs* 195.73% ± 11.22 (sucrose + bicarbonate), *p* = 0.006]; pendrin was also higher [100% ± 50.90 (sucrose) *vs* 326.03% ± 75.74 (sucrose + bicarbonate), *p* = 0.03]. Alkalosis-triggered K^+^ loss was confirmed in wild-type mice treated with vehicle (2% sucrose drinking water) or 0.28 M NaHCO_3_ (with 2% sucrose in drinking water) for 24 h (Genini et al., 2020) with 11.09 ± 4.62 μmol/body weight (BW) (g)/24 h for vehicle *vs* 22.93 ± 4.80 μmol/body weight (BW) (g)/24 h for NaHCO_3_, *p* = 0.008 (Figure 7C).

### Downregulation of Pendrin in KCC3-Knockout Mice

**FIGURE 8:**
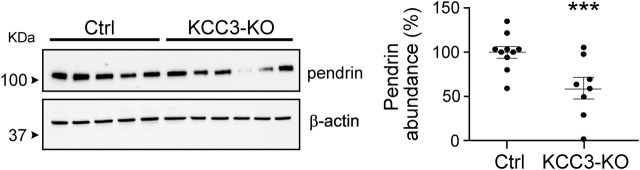
KCC3 deletion in KCC3-knockout (KCC3-KO) mice downregulated pendrin abundance compared to control (Ctrl), (two-tailed unpaired *t*-test). For blot quantification, densitometric values were normalized to β-actin. Values are means ± SEM; values in parentheses indicate n values. ***p < 0.001.

We evaluated pendrin abundance in KCC3-KO mice to confirm the functional linkage of KCC3a to pendrin and found that pendrin abundance was downregulated in KCC3-KO mice, implying that KCC3a is required for pendrin. Pendrin abundance was 100% ± 6.58 in control mice and 59.23% ± 11.92 in KCC3-KO mice, *p* = 0.006 (Figure 8).

## Discussion

Limited information exists on the expression and role of K–Cl cotransporters in the kidney. Immunofluorescence studies have localized KCC3 on the basolateral membrane of murine proximal tubule epithelial cells (Mercado et al., 2005) and KCC4 on the basolateral membrane of rabbit DCT (Velázquez and Silva, 2003) and mouse intercalated cells type-A (Boettger et al., 2002). A mathematical model of rat distal convoluted tubule addressed the presence of a K–Cl cotransporter along the basolateral membrane of the distal convoluted tubule and viewed it necessary for luminal Cl^−^ reabsorption and cell volume maintenance (Weinstein, 2005). The nature of the K–Cl cotransporter in mouse DCT is still unknown. In the Mercado study, the proximal tubule KCC3 expression was demonstrated using an exon 3–specific antibody (Mercado et al., 2005) which cannot discriminate between the two main isoforms, KCC3a and KCC3b. Since we demonstrated in 2001 that KCC3b was the major isoform in the kidney, whereas KCC3a was the predominant isoform in the brain (Pearson et al., 2001), it was likely that proximal tubule cells were expressing KCC3b. This is further evidenced in this study where we used a KCC3a-specific antibody and observed signal only in the distal tubule.

We provide here the first report of a K–Cl cotransporter expressed on the luminal membrane of renal epithelial cells. Indeed, we observed intense KCC3a-specific staining at the apical membrane of cells expressing the V-ATPase and pendrin, thus localizing KCC3a in type-B or type-nonA/nonB intercalated cells. Furthermore, the KCC3a-expressing cells were surrounded by calbindin-positive cells, restricting KCC3a expression to the CNT and cortical collecting duct (CCD), the aldosterone-sensitive distal nephron, which performs many key renal functions. In mice and rats, connecting tubules and cortical collecting ducts are composed of ∼40% intercalated cells and ∼60% principal cells. Pendrin-positive nonA/nonB cells are found in abundance in the CNT of the mouse kidney, while pendrin-positive type-B cells predominate in the CCD (Kim et al., 1999; Song et al., 2007).

Net transport through KCC3 is driven by the combined gradients of K^+^ and Cl^−^ across the membrane, and possibly the need for cell volume (swelling) activation. Microperfusion studies in the distal nephron provide luminal concentration values for K^+^ and Cl^−^ of ∼2 mM and 30–60 mM, respectively [summarized in Weinstein (2005)]. This means that the product of [K^+^] x [Cl^−^] on the outside is 20–25 times smaller than the product on the inside, and it would take an excess of 40–50 mM luminal K^+^ to start reversing the gradients. Thus, due to the high intracellular and low luminal concentration of K^+^ and Cl^−^, transport through apical KCC3 should be poised in the direction of secretion, that is, K^+^ loss.

Hydrochlorothiazide and amiloride are used as diuretics. Though both drugs trigger diuresis and a reduction in blood volume, they are known to have opposite effects on blood K^+^. The fact that both diuretics led to an increase in KCC3a abundance indicates that volume contraction rather than blood K^+^ was the factor that influenced the expression of the cotransporter. Similarly, long-term K^+^ depletion is known to result in nephrogenic diabetes insipidus and the production of a large amount of urine (Marples et al., 1996; Oksche and Rosenthal, 1998; Boyd-Shiwarski et al., 2020). In mice, a long-term K^+^-free diet also causes an increase in water excretion (Boyd-Shiwarski et al., 2020; Al-Qusairi et al., 2021 #4628). Plasma osmolarity, plasma Na^+^, and electrolyte-free water clearance are higher on K^+^-free diet than on a control K^+^ diet. Furthermore, the vasopressin surrogate marker, copeptin, was also increased on K^+^-free diet compared to the control diet (Al-Qusairi et al., 2021). As desmopressin treatment could not reverse the water diuresis in mice maintained on K^+^-free diet, the data suggested that low blood K^+^ is associated with nephrogenic diabetes insipidus. Diuresis and the fall in blood K^+^ occurred concomitantly after four days on K^+^-free diet. In our study, although the effect of the K^+^-free diet at the cellular level was not investigated, in line with the observation of hydrochlorothiazide and amiloride treatments, we observed increased KCC3a abundance in mice on K^+^-deficient diet.

Dehydration, imposed by water restriction, implies a reduction in total body water leading to hypertonicity. Dehydration is known to cause a reduction in body weight and mean arterial pressure, along with an increase in hematocrit, plasma osmolality, and vasopressin levels. To investigate the effect of total body water reduction on KCC3a, we performed a 23-h water deprivation study and observed that water-deprived mice also displayed a higher abundance of KCC3a. Na^+^ depletion leads to a reduction of the extracellular fluid compartment that is associated with a reduction in total body fluid (Hurley and Johnson, 2015). Consistently, we observed an increased abundance of KCC3a in mice exposed to a low-Na^+^ diet compared with a high-Na^+^ diet.

Contraction alkalosis is another possible reason of KCC3a upregulation caused by water loss. The extracellular fluid (ECF) volume reduces in contraction alkalosis with constant level of HCO_3_^−^. The ECF volume contraction causes alkalosis when stomach HCl is lost through vomiting or thiazide and/or loop diuretic treatment (Emmett, 2020). In our investigation, NaHCO_3_ treatment increased both pendrin and KCC3a abundance, whereas deleting KCC3 decreased pendrin abundance, suggesting that KCC3a and pendrin are functionally linked. Notably, other transporters have been associated with pendrin function. Alterations in the amount of pendrin have been linked to changes in the cystic fibrosis transmembrane conductance regulator, CFTR (Ko et al., 2002). CFTR likely aids in the recycling of Cl^−^ during pendrin activity. Although CFTR is also known to secrete HCO_3_^−^ (Kunzelmann et al., 2017; Berg et al., 2021), it is unknown whether CFTR mediates renal HCO_3_^−^ secretion, independent of pendrin. Alkalosis-induced upregulation of renal CFTR may result in direct HCO_3_^−^ secretion by CFTR channels along the distal nephron.

**FIGURE 9:**
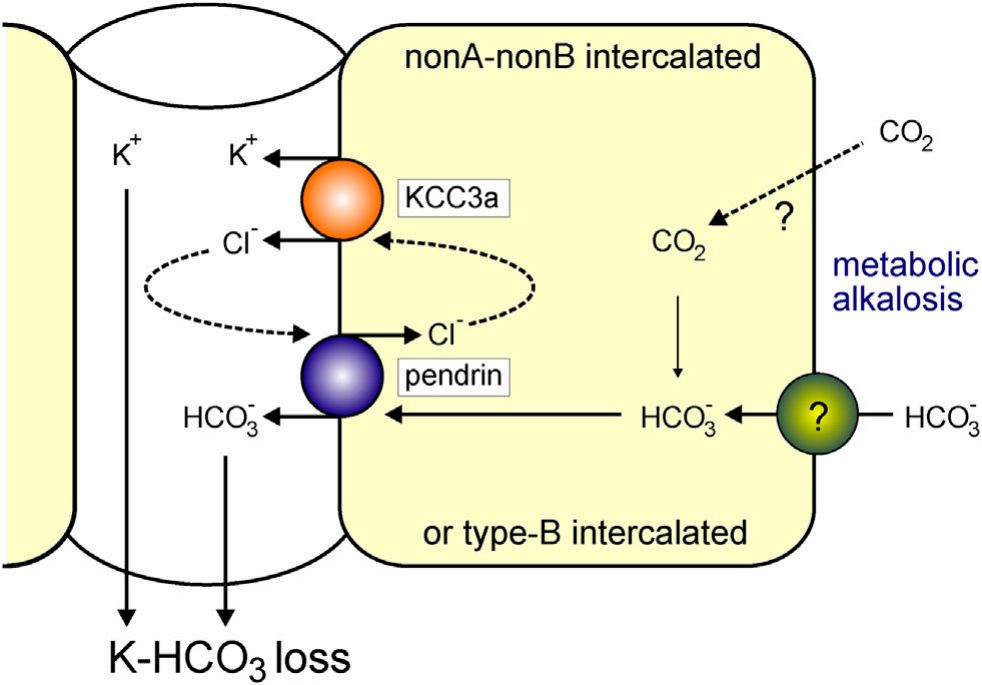
KCC3a participates in K^+^ loss in isolated alkalemia caused by metabolic alkalosis. Increased HCO_3_^−^ with a consequent increase in blood pH is known as alkalemia. NonA/nonB and/or type-B intercalated cells express pendrin and KCC3a at the apical membrane. Pendrin and KCC3a are upregulated during base loading or metabolic alkalosis. As KCC3a secretes K^+^ and Cl^−^ and pendrin recycles Cl^−^ in exchange for HCO_3_^−^, this leads to a net loss of KHCO_3_.

Acute metabolic alkalosis prevents the proximal tubule from reabsorbing NaHCO_3_ and fluid, resulting in increased distal delivery of Na^+^ and HCO_3_^−^, which promotes K^+^ secretion by the distal nephron (Malnic et al., 1972). Greater distal Na^+^ and fluid supply, as well as a luminal or systemic increase in HCO_3_^−^ level and pH, stimulate ENaC, ROMK, and BK, resulting in increased distal K^+^ secretion (Khuri et al., 1975; Palmer et al., 1998; Satlin et al., 2006; Pech et al., 2010). Increased K^+^ secretion *via* ROMK would ultimately lessen the driving force for K^+^ secretion by hyperpolarizing the membrane potential (Huang and Kuo, 2007). This would ultimately restrict overall K^+^ secretion. Given the foregoing, KCC3a appears to be a likely candidate for the K^+^ secretion observed during metabolic alkalosis. KCC3a most likely acts as a recycling mechanism for the Cl^−^ ions that pendrin needs to secrete HCO_3_^−^ into the filtrate. A similar role has been assigned to the sodium-driven chloride/bicarbonate exchanger (NDCBE), which facilitates the recycling of Cl^−^ and HCO_3_^−^. However, NDCBE is required to maintain sodium balance and intravascular volume during salt depletion (Sinning et al., 2017). Moreover, pendrin is also more abundant and active in nonA and nonB intercalated cells (Kim et al., 2002) that do not express NDCBE (Roy et al., 2015). As KCC3a expression overlaps with pendrin expression, the cotransporter could potentially play an important role in Cl^−^ recycling in nonA/nonB intercalated cells, allowing pendrin to secrete HCO_3_^−^ into the tubular lumen. This function would result in net KHCO_3_ secretion (Figure 9). In metabolic alkalosis, the kidney seems then to prioritize bicarbonate secretion over K^+^ loss. In a recent study, Wall et al. reported that pendrin-KO mice excreted more K^+^ than wild-type mice on a combined NaCl- and K^+^-deficient diet (Pham et al., 2022). While pendrin-KO mice have normal acid/base balance under basal conditions (Kim et al., 2005), they develop severe metabolic alkalosis due to combining NaCl- and K^+^-deficient diet. We found that KCC3a was upregulated in response to metabolic alkalosis produced by NaHCO_3_ administration and on both NaCl- and K^+^-deficient diets. Based on our current findings, the observed increased K^+^ excretion in pendrin-KO mice on a combined NaCl- and K^+^-deficient diet could be attributable to the upregulation of KCC3a in pendrin-KO mice.

## Data Availability

The raw data supporting the conclusion of this article will be made available by the authors, without undue reservation.
